# Modelling and predicting the effect of social distancing and travel restrictions on COVID-19 spreading

**DOI:** 10.1098/rsif.2020.0875

**Published:** 2021-02-10

**Authors:** Francesco Parino, Lorenzo Zino, Maurizio Porfiri, Alessandro Rizzo

**Affiliations:** ^1^ Department of Electronics and Telecommunications, Politecnico di Torino, 10129 Turin, Italy; ^2^ Faculty of Science and Engineering, University of Groningen, 9747 AG Groningen, The Netherlands; ^3^ Department of Mechanical and Aerospace Engineering, New York University Tandon School of Engineering, Brooklyn, NY 11201, USA; ^4^ Department of Biomedical Engineering, New York University Tandon School of Engineering, Brooklyn, NY 11201, USA; ^5^ Center for Urban Science and Progress, New York University Tandon School of Engineering, Brooklyn, NY 11201, USA; ^6^ Office of Innovation, New York University Tandon School of Engineering, Brooklyn, NY 11201, USA

**Keywords:** calibration, epidemic model, meta-population, mobility, networks, non-pharmaceutical interventions

## Abstract

To date, the only effective means to respond to the spreading of the COVID-19 pandemic are non-pharmaceutical interventions (NPIs), which entail policies to reduce social activity and mobility restrictions. Quantifying their effect is difficult, but it is key to reducing their social and economic consequences. Here, we introduce a meta-population model based on temporal networks, calibrated on the COVID-19 outbreak data in Italy and applied to evaluate the outcomes of these two types of NPIs. Our approach combines the advantages of granular spatial modelling of meta-population models with the ability to realistically describe social contacts via activity-driven networks. We focus on disentangling the impact of these two different types of NPIs: those aiming at reducing individuals’ social activity, for instance through lockdowns, and those that enforce mobility restrictions. We provide a valuable framework to assess the effectiveness of different NPIs, varying with respect to their timing and severity. Results suggest that the effects of mobility restrictions largely depend on the possibility of implementing timely NPIs in the early phases of the outbreak, whereas activity reduction policies should be prioritized afterwards.

## 1. Introduction

Following the first report of the novel coronavirus (SARS-CoV-2) in Wuhan, China, COVID-19 has risen above 83 million cases and 1 831 703 reported deaths as of 3 January 2021 [1]. The ongoing pandemic quickly reached Europe during February and March 2020, forcing most of the countries to implement unprecedented non-pharmaceutical interventions (NPIs) to fight the spread [2–6]. Some of these interventions promote policies to reduce human-to-human interactions, for example by enforcing social distancing, halting non-essential activities, closing schools and banishing large gatherings [2,5,6]. Others limit human mobility by means of travel restrictions and bans [6,7]. Owing to the considerable economic and social cost associated with the implementation of both of these types of policies [8–10], it is crucial to assess their effectiveness. Mathematical and computational epidemic models are key to being able to accurately evaluate a wide range of what/if scenarios and to predict the evolution of the pandemic for different choices of NPIs [7,11–18].

One of the fundamental aspects of the spread of infectious diseases is its spatial diffusion and the concurrent role of human mobility patterns [19–21]. Extensive studies on mobility within the COVID-19 pandemic revealed that population movements are among the main drivers of the spatial spreading of the outbreak [4,22]. Network structures have emerged as a powerful framework to encapsulate such mobility patterns within mathematical models of epidemics, especially by means of meta-population models [23]. This modelling paradigm is based on the definition of a set of communities (provinces, counties or regions), connected by a network that captures daily short-range commuting and long-range mobility.

Different from most of the classical meta-population models that tend to assume homogeneous mixing within each community [23,24], we propose a network structure that accounts for the inherent, heterogeneous and time-varying nature of human interactions [25,26], together with behavioural changes in response to the evolution of the pandemic [27,28]. To this aim, individuals interact on the basis of a mechanism inspired by activity-driven networks (ADNs) [29,30]. Our model includes two key aspects of social communities: mobility patterns and temporal, heterogeneous networks of contacts. Within this meta-population model, we incorporate a variation of a susceptible–infected–removed (SIR) epidemic process [31], which is designed to capture several key features of COVID-19, such as the presence of latency periods and the delay in the official reporting of infections and deaths.

We calibrate the model on epidemic data from the first wave of the Italian COVID-19 outbreak [32] to examine different scenarios that evaluate the spatial effects of NPIs. In particular, we explore the interplay between a reduction in social activity and mobility restrictions. At the modelling level, the former mechanism acts upon the network of contacts, while the latter modifies mobility patterns between communities. Our findings reveal that the timing of the interventions is essential for their effective implementation. We conclude that mobility restrictions should be applied at the early stage of the epidemic and coupled with appropriate policies to reduce social activity. Surprisingly, the impact of mobility restrictions is spatially heterogeneous. For the Italian outbreak, this results in a greater benefit for southern regions, that is, those located far from the initial outbreak. The overall effect of early travel restrictions in these areas led to a 12% reduction in the total number of deaths. We also examine different interventions across age cohorts, determining that the application of severe restrictions only to the most vulnerable age cohorts would not be sufficient to effectively reduce the death toll. Different phenomena are observed upon the relaxation of containment measures, with the contribution of continued mobility restrictions being negligible. In this phase of the fight against the epidemic, policies limiting social activity (for instance, by enforcing the use of face masks or social distancing) yield the main benefits in mitigating resurgent outbreaks.

## 2. Methods

### 2.1. Model

#### 2.1.1. Meta-population activity-driven model

We consider a population of *n* individuals partitioned into a set H={1,…,K} of communities, located in bounded geographical areas (administrative divisions, such as regions, provinces or municipalities), where *n*_*h*_ is the number of individuals in the *h*th community. Communities are connected through a weighted graph that models travel paths between them. The weight matrix *W* ∈ [0, 1]^*K*×*K*^, called the *routing matrix*, is a matrix with non-negative entries, zeros on the main diagonal and row sums equal to 1, such that *W*_*hk*_ is the fraction of members of community *h* that move to community *k* per unit-time.

**Figure 1.**
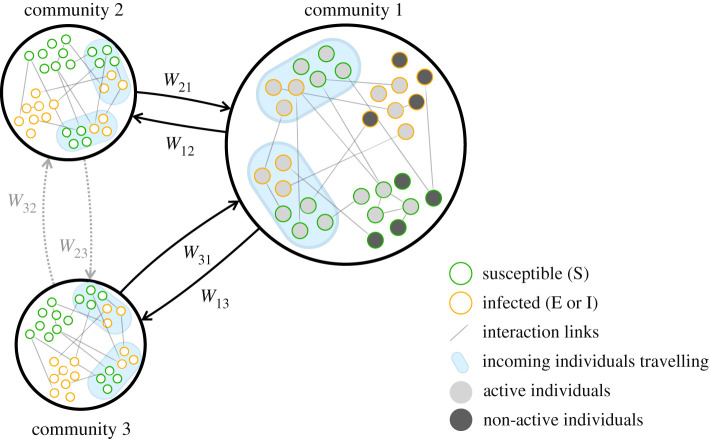
Schematics of the meta-population model, which illustrates the community structure and the role of the routing matrix *W*.

Individuals interact according to a mechanism inspired by ADNs [29,30], which accounts for the inherent, heterogeneous propensity of humans to interact with others. Specifically, individuals are divided into *P* baseline activity classes 0 < *a*_1_ < *a*_2_ < · · · < *a*_*P*_ ≤ 1, where the *baseline activity* *a*_*i*_ of individuals in the *i*th class quantifies their nominal propensity to interact with others. The latter can be interpreted as the average probability for an individual belonging to the *i*th class to generate interactions in a unit time step. At each time step and for each activity class *i*, a fraction *a*_*i*_ of individuals, selected uniformly at random, activates and generates interactions with others, regardless of their class. This fraction of the population is called *active*. Active individuals may generate interactions within the community where they are located, or they may travel and interact in other communities, before returning to their community, at the end of the time step. This aspect is modelled through a *mobility parameter* *b* ∈ [0, 1], which quantifies the baseline fraction of the active population that commutes to other communities; the commuting unfolds according to the routing matrix *W* (figure 1). The remaining fraction 1 − *b* of the active population does not commute and interacts locally with individuals randomly selected within their community. We assume that the *P* activity classes are equally distributed in different communities. Specifically, we introduce the activity distribution vector *η* ∈ [0, 1]^*P*^, such that the number of individuals with activity class *a*_*i*_ in the *h*th community is equal to the product *n*_*h*_*η*_*i*_. We finally introduce a parameter *m* ≥ 0 that captures the average number of contacts generated by each active individual in a time unit.

Two parameters, *α* ∈ [0, 1] and *β* ∈ [0, 1], are introduced to model NPIs. The former, *α*, models individuals’ self-isolation owing to the awareness of the disease spreading. In the model, this corresponds to scaling down the individual activity from its baseline value *a*_*i*_ to *αa*_*i*_. The latter, *β*, captures the effect of mobility restrictions, which are modelled by scaling down the baseline mobility parameter from *b* to *βb*.

#### 2.1.2. Disease progression

**Figure 2.**
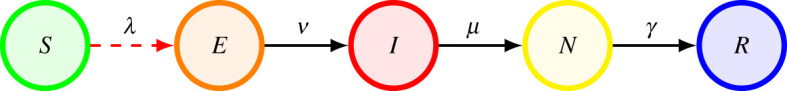
Schematic of the epidemic progression. Individuals may be susceptible to the disease (*S*), exposed but yet not infectious (*E*), infectious (*I*), non-infectious (*N*) and recovered or dead (*R*). The compartment *N* captures the delay between the end of the infectious period and the reporting of a death, and is key for parameter identification from real-world data. All the transition rates are detailed in the main text and reported in table 1.

The disease progression is modelled according to an extension of the classical SIR model (figure 2), which encapsulates a latency period between contagion and infectiousness, a limited duration of the infectious period, coinciding with the peak of the viral load, and a delay for deaths reporting [2]. Specifically, we adopt a susceptible–exposed–infectious–non-infectious–removed (SEINR) compartmental model (figure 2). After contagion, infected individuals become initially exposed (*E*) before spontaneously moving into the infectious (*I*) compartment with rate *ν*. Once the infectious period terminates (with rate *μ*), individuals transition to the non-infectious compartment (*N*), before recovering (or dying) with rate *γ*, which is represented by the *R* compartment. The compartment *N* captures the delay between the end of the infectious period and the reporting of a death. The number of deaths is the most reliable parameter for calibration, given the uncertainty in reporting active infectious cases. The parameters have immediate interpretation: 1/*ν* is the average latency period of the disease (time from contagion to infectiousness), 1/*μ* is the average period of communicability (in which individuals are infectious) and 1/*γ* captures the delay before reported deaths. Hence, 1/*μ* + 1/*γ* is the average time from infectiousness to the reported death. We comment that other important features of COVID-19 may be easily incorporated by considering further compartments into the model, similar to [33]. In this vein, one may include, for instance, a differentiation between symptomatic and asymptomatic infectious individuals, which might help implement timely feedback control interventions.

**Table 1.** Model parameters; parameters indicated with a tick (✓) are identified by fitting real-world data of reported deaths from [32]. From the table, we derive the following parameters: $\nu =0.156\;d^{-1}$, $\mu =0.2\;d^{-1}$ and $\gamma =0.105\;d^{-1}$.

	meaning	value(s)	reference
1/*ν*	latency period	6.4 days	[2,43]
1/*μ*	infectiousness period	5 days	[2,44]
1/*γ*	time from infectiousness to reported death	9.52 days	[2,44,45]
*λ*	per-contact infection probability	0.042	✓
*η*	class distribution	[0.768, 0.232]	[39,40]
*a*	baseline activity	[0.149, 0.545] d^−1^	[40]
*b*	mobility parameter	0.09	[39]
*α*_low_	activity reduction	0.176	✓
*m*	average number of contacts	19.77	[40]
*β*_low_	mobility reduction	0	✓

The contagion mechanism involves an interaction. We introduce a parameter *λ* ∈ [0, 1], which captures the fraction of susceptible individuals who become exposed after an interaction with an infectious individual. The contagion depends not only on *λ*; it depends also on individual properties (their activity) and on the network structure, as well as on the prevalence of infectious individuals. We denote by $\Pi_{i}^{h}(t)$ the *contagion function*, that is, the fraction of susceptible individuals with activity *a*_*i*_ who belong to community *h* and who become infected at time *t*, whose expression is detailed in the following.

#### 2.1.3. Dynamics

We consider the generic activity class *i* and community h∈H. Let $S_{i}^{h}$, $E_{i}^{h}$, $I_{i}^{h}$ and $N_{i}^{h}$ be macroscopic variables counting the number of susceptible, exposed, infectious and non-infectious individuals of class *i* in community *h*, respectively. Clearly, $n_{h}\eta_{i}-S_{i}^{h}-E_{i}^{h}-I_{i}^{h}-N_{i}^{h}$ is the number of removed individuals in class *i* and community *h*. In the thermodynamic limit of large populations [23,34,35], *n* → ∞, we describe the epidemic spreading in terms of the macroscopic variables by writing the following system of mean-field recurrence equations:

2.1 $$\begin{array}{rl} & S_{i}^{h}(t+1)=(1-\Pi_{i}^{h}(t))S_{i}^{h}(t),\\ & E_{i}^{h}(t+1)=\Pi_{i}^{h}S_{i}^{h}(t)+(1-\nu )E_{i}^{h}(t),\\ & I_{i}^{h}(t+1)=\nu E_{i}^{h}(t)+(1-\mu )I_{i}^{h}(t),\\ \;\;\; & N_{i}^{h}(t+1)=\mu I_{i}^{h}(t)+(1-\gamma )N_{i}^{h}(t). \end{array}\}$$

We now detail the contagion mechanism and derive an explicit expression for $\Pi_{i}^{h}(t)$. To simplify the notation, we omit time *t*, that is, $\Pi_{i}^{h}$ is used to denote $\Pi_{i}^{h}(t)$. In the thermodynamic limit of large populations *n* → ∞ and assuming that the epidemic prevalence is small (so that we can neglect the probability of having multiple interactions with infectious individuals at the same time), the quantity $\Pi_{i}^{h}$ can be written as the sum of four different terms. The first summand accounts for the contagions caused by the fraction *αa*_*i*_(1 − *βb*) of active individuals from $S_{i}^{h}$ who remain in the *h*th community and who interact therein with infectious individuals; the second summand accounts for the infections caused by the fraction (1 − *αβa*_*i*_*b*) of $S_{i}^{h}$ who remain in community *h* and who come into contact therein with active infected individuals; the third and the fourth summands account for the contagions of the fraction *αβa*_*i*_*b* of $S_{i}^{h}$ who are active and who move to other communities, interacting with infected individuals or receiving interactions from active infectious individuals in the community they move to, respectively. These four terms yield

2.2 $$\begin{array}{rl} \Pi_{i}^{h} & =m\alpha a_{i}(1-\beta b)\lambda P_{h}+m(1-\alpha \beta a_{i}b)\lambda Q_{h}\\ & \;+m\alpha \beta a_{i}b\sum_{k\in H}W_{hk}\lambda P_{k}+m\alpha \beta a_{i}b\sum_{k\in H}W_{hk}\lambda Q_{k}, \end{array}$$

where

2.3a $$P_{h}=\frac{1}{{\tilde{n}}_{h}}(\sum_{\;j=1}^{P}(1-\alpha \beta a_{\;j}b)I_{\;j}^{h}+\sum_{k\in H}W_{kh}\sum_{\;j=1}^{P}\alpha \beta a_{\;j}bI_{\;j}^{k})$$

and

2.3b $$Q_{h}=\frac{1}{{\tilde{n}}_{h}}(\sum_{\;j=1}^{P}(1-\beta b)\alpha a_{\;j}I_{\;j}^{k}+\sum_{k\in H}W_{kh}\sum_{\;j=1}^{P}\alpha \beta a_{\;j}bI_{\;j}^{k})\;$$

are the fractions of infectious and active infectious individuals who are present in community *h*, respectively. The quantity

2.4 $${\tilde{n}}_{h}=(1-\alpha \beta \langle a\rangle b)n_{h}+\alpha \beta \langle a\rangle b\sum_{k\in H}W_{kh}n_{k}$$

is the number of individuals who are located in community *h*, where $\langle a\rangle :=\sum_{i=1}^{P}\eta_{i}a_{i}$ is the average activity of the population.

### 2.2. Model calibration

We calibrate the model to reproduce the COVID-19 outbreak in Italy, setting provinces as communities, using epidemiological parameters from the medical literature [36–38], mobility data from the Italian National Institute of Statistics (ISTAT) [39] and data from officially reported deaths [32]. Based on available empirical data on social contacts per age group [40], we partition the population into two activity classes. The population below 65 years old forms the *high-activity class*, and the population above 65 years old constitutes the *low-activity class*. Different mortality rates are associated with the two classes to estimate the number of deaths, based on serology-informed data [41]. Practically, the high-activity class may encompass the low-risk/high-mobility portion of the population, and the low-activity class may comprise the high-risk/low-mobility portion of the population. The details of the model calibration are given below.

#### 2.2.1. Calibration of the meta-population model

The Italian territory is divided into *K* = 107 provinces, which are selected as the metapopulation model communities, extracting the corresponding population *n*_*h*_ from the census data [39]. Provinces are administrative units that offer most of the essential services to the population (supermarkets, hospitals, schools, public offices, restaurants, etc.). Hence, our choice of spatial granularity allows one to distinguish and disentangle the effects of the two categories of NPIs considered in this paper, whereby activity reduction refers to the execution of everyday-life activities within a province, and mobility restrictions prevent travel between provinces. Provinces are grouped into 20 regions, gathered in five macro-regions: *north-west*, *north-east*, *centre*, *south* and *islands* (electronic supplementary material, section S1 and figure S1).

We partition the population into *P* = 2 activity classes, based on age-stratified data on social contacts [40], aggregating age groups that form a high or low number of contacts, respectively. Specifically, the former contains people below 65 years old, while the latter contains people above 65 years old. According to the same study, we set *m* = 19.77. The baseline activity classes *a*_1_ and *a*_2_ are determined by matching the average number of contacts of the individuals in the classes. The fraction *η*_*i*_ of population in each class is determined from the Italian age distribution [39]. Simulations are presented in the electronic supplementary material, figure S8 to show the robustness of our results for different levels of class partitioning.

**Figure 3.**
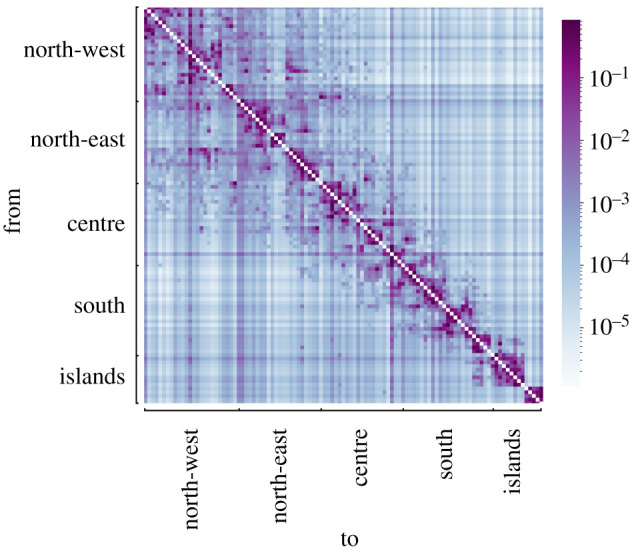
Heat map representing the routing matrix *W* between provinces estimated from [39]. The colour code represents the fraction of active people who travel from one province to another. Provinces are gathered in macro-regions, as detailed and illustrated in the electronic supplementary material, §S1 and figure S1.

We consider two types of mobility: the commuting pattern between provinces and long-range mobility. The former is directly obtained from the 2011 census data in the ISTAT database [39], which has been validated and adopted to model mobility in recent works on COVID-19 [13]. Comprehensive data on long-range mobility are not available. We estimate them as follows. For each province, we consider the number of nights spent in accommodation facilities over the period from February to May 2011, which represents the destinations of travellers [39]. Origins are estimated based on the flows between macro-regions [39]. Assuming uniformity within each macro-region, we set the origins proportional to the population of each province. Finally, *W* is obtained by combining the two origin–destination matrices (figure 3). The mobility parameter *b* is estimated as the fraction of the population who move outside their province, using data from [39].

NPIs are implemented as follows. At *t* = *t*_0_, we set *α* = *β* = 1. Then, based on empirical data [42], we consider a linear decrease over 15 days to reach a value *α*_low_. Such a decrease begins on 5 March (the day of the enforcement of the first social distancing measures) and ends on 20 March (when a severe lockdown is enacted). Similarly, *β* is reduced to *β*_low_. As suggested in [42], mobility restrictions have not been implemented uniformly county wide: changes were enforced on 1 March for macro-regions *north-west*, *north-east* and *centre*, and on 7 March for *south* and *islands*. The values of *α*_low_ and *β*_low_ are identified from epidemic data.

#### 2.2.2. Calibration of the epidemic parameters

Epidemic parameters are taken from the literature on COVID-19. Specifically, the latency period 1/*ν* and the infectious period 1/*μ* are taken from [2], based on clinical estimations from [43,44], respectively; *γ* is the inverse of the difference between the average time from infectiousness to the reported death [45] and 1/*μ*. The infection probability *λ* depends on the model of social interactions. Hence, we identify it from real-world data. Table 1 reports the parameters used in our simulations.

#### 2.2.3. Parameter identification

We calibrated our model by fitting the temporal evolution of the reported deaths, during the COVID-19 outbreak in Italy. Data at the regional level were retrieved from the official Italian Dipartimento della Protezione Civile [32] database. This database starts on 24 February, and we had extended it backwards in time for 20 days (until 4 February). We filled with zero deaths the section of the database from 4 February to 20 February, and we manually corrected the database to include seven deaths that were not reported therein in the period from 20 to 23 February (electronic supplementary material, section S2). To calibrate the model, we focused on the period from 4 February (denoted as *t*_0_) to 18 May (denoted as *t*_end_); namely, until the first relaxation of NPIs. To enhance the reliability of the data, we applied a weekly moving average.

Using the SEINR epidemic progression model, we computed the deaths in province *h* for activity class *i* as a fraction of the removed individuals $R_{i}^{h}(t)$, according to the class fatality ratio $f_{1}=0.045\text{\%}$ and $f_{2}=5.6\text{\%}$. The latter was inferred from a serology-informed estimate performed on age-stratified data from Geneva, Switzerland [41], scaled on the Italian age distribution using census data [39]. Since we had no access to reliable information about the initial number of exposed $E_{i}^{h}(t_{0})$ or infected $I_{i}^{h}(t_{0})$ individuals, such initial conditions needed to be identified. For each province *h* and activity class *i*, we initialized the number of exposed and infected as a fraction *k*_1_ and *k*_2_ of the total reported cases at the end of the observation time (24 June) *C*^*h*^(*t*_end_) from the official database [32],

2.5 $$E_{i}^{h}(t_{0})=k_{1}\eta_{i}C^{h}(t_{\mathrm{end}})\;\mathrm{and}\;I_{i}^{h}(t_{0})=k_{2}\eta_{i}C^{h}(t_{\mathrm{end}}),$$

where *k*_1_ and *k*_2_ were identified together with the other parameters.

The parameter identification was formulated as a minimization problem, solved by means of a dual-annealing procedure [46]. Specifically, we defined the cost function *c* as the weighted sum of the squared error between the number of deaths predicted by the model and the regional real data, normalized with respect to the maximum number of deaths in the region. To this aim, we defined the set of regions R and the partition of provinces into regions as the function π : H→R, such that *π*(*h*) = *r* if and only if province *h* was located in region *r*. For each r∈R, we introduced

2.6 $$c^{r}=\sum_{t=t_{0}}^{t_{\mathrm{end}}}{\left(\frac{D_{\mathrm{MA}}^{r}(t)}{{\bar{D}}_{\mathrm{MA}}^{r}}-\frac{\sum_{h:\pi (h)=r}fR^{h}(t)}{{\bar{D}}_{\mathrm{MA}}^{r}}\right)}^{2},$$

and the cost function as the sum of *c*^*r*^ weighted with the total number of deaths in the region *r* is

2.7 $$c=\sum_{r\in R}\left(c^{r}\sum_{t=t_{0}}^{t_{\mathrm{end}}}D^{r}(t)\right).$$

**Figure 4.**
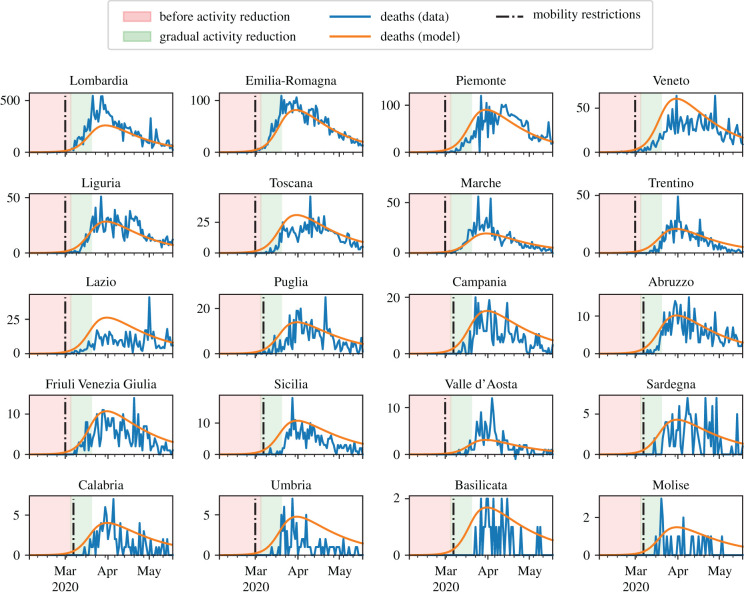
Results of the model calibration aggregated at the region level. Model parameters are summarized in table 1. The red area denotes the time interval before the implementation of activity reduction. The green area denotes the 15 days in which *α* decreases linearly from 1 to *α*_low_ = 0.176. In the white area, activity is reduced to *α*_low_. The black, dash-dotted vertical line denotes the implementation of mobility restrictions. Orange curves illustrate the predictions from the model; blue curves are actual deaths data from [32].

Here, *D*^*r*^(*t*) indicates the reported deaths in region *r*, $D_{\mathrm{MA}}^{r}(t)$ is the weekly moving average and ${\bar{D}}_{\mathrm{MA}}^{r}=\max_{t}D_{\mathrm{MA}}^{r}(t)$ is its maximum value. Using the fatality ratio, the model predicts *f R*^*h*^(*t*) deaths in province *h* at time *t*. Figure 4 shows the real and simulated time series with the identified parameters.

## 3. Results

### 3.1. Implementation of NPIs

Here, we elucidate the role of NPIs in halting the spread of COVID-19. We aim at disentangling the contribution of the two most common kinds of interventions: reduction of individuals’ activity, through lockdown or social distancing, and enforcement of mobility restrictions. We take as a reference the NPIs implemented in Italy (detailed in the electronic supplementary material, §S2) and identify the NPI-related parameters from available data. The enforcement of lockdown and social distancing policies, gradually enacted during a time window of two weeks (from 5 to 20 March), are modelled through a linear decrease of the *α* parameter from 1 to *α*_low_ = 0.176. The effect of the nearly complete mobility restrictions between provinces can be seen from 1 March in the northern macro-regions and from 7 March in the southern ones [42]. We model these restrictions by setting the mobility parameter to *β*_low_ = 0 on the corresponding dates.

**Figure 5.**
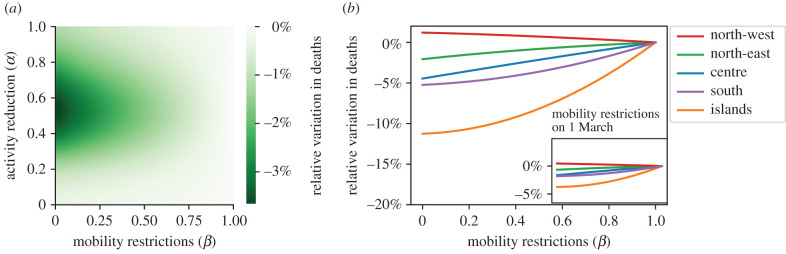
Effect of early application of mobility restrictions. We consider the total number of deaths over a time window of 104 days from the beginning of the simulations (4 February) to the time corresponding to the relaxation of the most severe NPIs in Italy (18 May). In (*a*), we illustrate the interplay between the two NPI mechanisms, assuming an earlier application of mobility restrictions and activity reduction applied at the original date (5 March). We consider different combinations of levels of activity reduction and mobility restrictions, where higher levels of *α* or *β* denote less severe NPIs. The heat map codes the reduction in deaths with respect to a scenario where mobility restrictions are not applied. (*b*) The effect of earlier mobility restrictions at a macro-regional level, assuming a level of activity reduction as per the lockdown phase (*α*_low_ = 0.176). The inset illustrates the effect corresponding to the application of the same restrictions at the actual date (1 March).

We start by investigating the effect of mobility restrictions in combination with activity reduction policies (figure 5*a*). We simulate the mobility restrictions as being applied on 4 February, that is, almost one month earlier than the actual date. We compare the number of deaths over the time window that ranges from 4 February to the date of the first relaxation of NPIs in Italy (18 May). We observe that the effect of mobility restrictions becomes significant for intermediate levels of activity reduction policies (that is, 0.3 < *α* < 0.7). On the other hand, a negligible effect of mobility restrictions is registered for milder levels of activity reduction policies (*α* > 0.7) and for extremely severe activity reductions (*α* < 0.3). The latter, counterintuitive, finding is due to a balance between the increased number of deaths in some northern macro-regions (close to the initial outbreak) and the decrease in deaths in others (electronic supplementary material, figure S5).

To detail this mechanism, we examine the number of deaths in each macro-region, using different levels of mobility restrictions and setting the activity reduction to the lockdown level, *α*_low_ = 0.176 (figure 5*b*). Our results suggest that the impact of mobility restrictions is strongly dependent on their geographical location, and it vanishes if it is not implemented in a timely way. Notably, we find that the timely implementation of severe mobility restrictions would have reduced the number of deaths by more than 12% in the *islands* macro-region (that is, far from where the outbreak was initially located) over the duration of severe NPIs. Such an advantage becomes smaller and smaller as the considered macro-regions are closer to the initial location of the outbreak. Paradoxically, mobility restrictions even become slightly detrimental if applied in the *north-west* macro-region, where the outbreak started. This is due to the commute of infected individuals from the most affected provinces to the rest of the provinces and of susceptible individuals from less impacted provinces to the rest of the country. For comparison, we also report the death count for the implementation of the same restrictions on 1 March, which corresponds to the actual date of their implementation. We observe that the timing of NPIs is essential; an earlier application of travel restrictions by one month would have saved twice as many lives. Similar results are obtained for the peak of the epidemic incidence (electronic supplementary material, figures S6 and S7).

**Figure 6.**
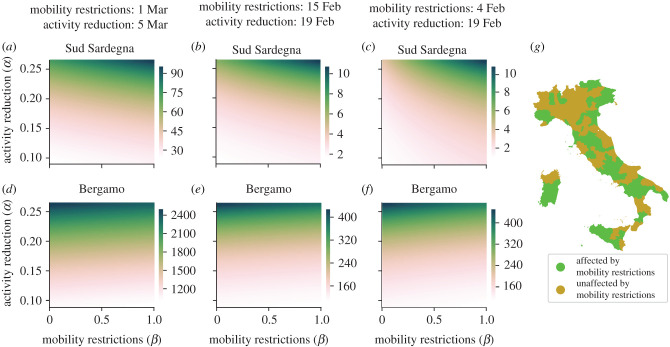
Effect of activity reduction and mobility restrictions, where higher levels of *α* or *β* denote less severe NPIs. We consider the total number of deaths over a time window of 104 days from the beginning of the simulation (4 February) to the time corresponding to the relaxation of the most severe NPIs in Italy (18 May). We investigate three different intervention scenarios. In panels (*a*,*d*), both mobility restrictions and activity reduction are applied on the actual application dates. In panels (*b,e*), both strategies are hypothetically implemented earlier by 15 days. In panels (*c*,*g*), mobility restrictions are further set earlier on 4 February, while keeping activity reduction applied earlier by only 15 days. In panels (*a*–*c*), the province of *Sud Sardegna* is shown as an example of the positive effect of early mobility restrictions. In panels (*d*–*f*), the province of *Bergamo* appears to be unaffected by mobility restrictions. In (*g*), we illustrate the classification of the provinces as affected or unaffected by mobility restrictions, considering the scenarios relative to panels (*c*,*f*).

The large geographical variability in the number of deaths is confirmed in figure 6*a*–*f* , which shows the total number of deaths for two representative provinces under different timing and intensity of implementation of NPIs. While the province of *Bergamo* (in the *north-west* macro-region), which had one of the earliest and biggest outbreaks, seems unrelieved by mobility restrictions, the province of *Sud Sardegna* (in the *islands* macro-region), an area much less affected by the pandemic than the former, would have largely benefited by such an intervention. To investigate this further, we factor out the role of the two types of NPIs by performing a non-negative matrix factorization [47] on the outcome of our simulations at the province level (details in the electronic supplementary material, §§S3 and S4). Specifically, we focus on values of *α* ranging over a ±50% interval with respect to the value of the lowest activity coefficient *α*_low_ = 0.176, identified from real-world data during the lockdown, and we simulate the early application of mobility restrictions with different intensity levels and timing. Our analysis leads to the characterization of two sets of Italian provinces. The first (in green in figure 6*g*) comprises provinces where timely implemented mobility restrictions are effective in reducing epidemic prevalence (for example, *Sud Sardegna*). The second set (in brown in figure 6*g*) contains provinces for which mobility restrictions have instead a negligible impact. Predictably, most of the provinces in the *north-west* (where the outbreak started) are unaffected by mobility restrictions, while the majority of provinces in the *south* and *islands* would have benefited from an earlier implementation of such restrictions.

Surprisingly, some important exceptions are identified. For instance, the provinces of *Varese* and *Monza* (close to the Milan metropolitan area) would have benefited from timely mobility restrictions. We believe that this is due to the initially small number of cases in those two provinces, and to the large number of daily commuters from those provinces to the *Milan* province and other neighbouring locations, where the Italian outbreak started. Hence, the same dynamics between north and south Italy is documented again over a much smaller spatial scale, between northern provinces with a larger initial difference in epidemic prevalence. Similar results are observed for other intervention scenarios (electronic supplementary material, figure S2).

**Figure 7.**
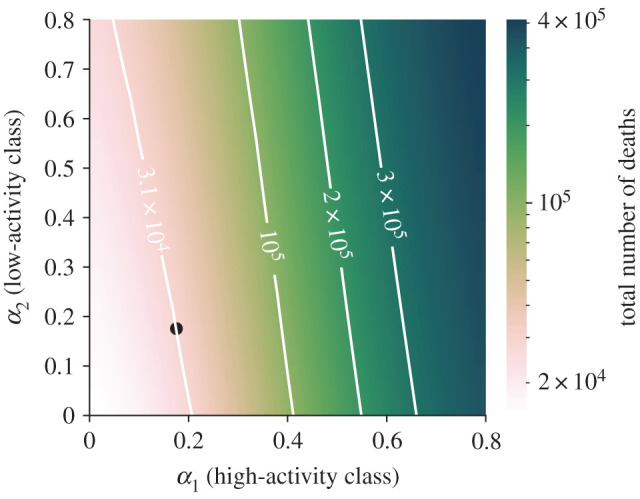
Effect of targeted lockdown strategies. We consider the total number of deaths over a time window of 104 days from the beginning of the simulation (4 February) to the time corresponding to the relaxation of the most severe NPIs in Italy (18 May). On the two axes, *α*_1_ and *α*_2_ correspond to activity reduction in high- and low-activity classes, respectively, where higher levels of *α* denote less severe NPIs. Level curves are shown to clarify how targeted interventions should be combined to produce the same effect on the total number of deaths. The black dot represents the identified activity reduction in model calibration.

Finally, we discuss the possibility of implementing targeted activity reductions that act independently on the two activity classes. This allows us to study the effectiveness of differential intervention policies that could aim at strongly reducing social activity for age cohorts that are more at risk of developing severe illness, while implementing mild restrictions for younger people. Instead of a single parameter *α*, we thus introduce two parameters *α*_1_ and *α*_2_ that measure the activity reduction for the high- and the low-activity classes, respectively. The heat map in figure 7 illustrates the effect of different combinations of *α*_1_ and *α*_2_ on the total number of deaths; the level curves help us to understand the trade-off in targeting the two classes. We observe that the total number of deaths is mostly determined by the parameter *α*_1_, that is, the activity reduction for the high-activity class. Hence, our results suggest that implementing targeted stay-at-home policies in which severe activity reductions are only enforced on the age cohorts that are more at risk (in our scenario, people over 65 years old) is not sufficient to reduce the overall death toll.

### 3.2. Relaxation of NPIs towards reopening strategies

The proposed meta-population model enables the analysis of reopening strategies to relax restrictions while avoiding resurgent outbreaks. This has recently emerged as a key issue in the control of COVID-19 outbreaks in the medium- to long-term period [48]. We run our calibrated model to simulate the epidemic until the date of intervention relaxation. Then, we vary the values of parameters *α* and *β* to account for the relaxation of the containment measures. Similar to the previous analysis, we consider a set of different options for the parameters after intervention relaxation and different times for starting the reopening strategies. Specifically, we model the relaxation of the reduction of social activity by varying the parameter *α* from *α*_low_ , identified during the lockdown, to a value of *α* = 0.6. Likewise, we describe the uplifting of mobility restrictions by varying the parameter *β* from 0 (no mobility allowed) to 1 (nominal mobility reinstated).

**Figure 8.**
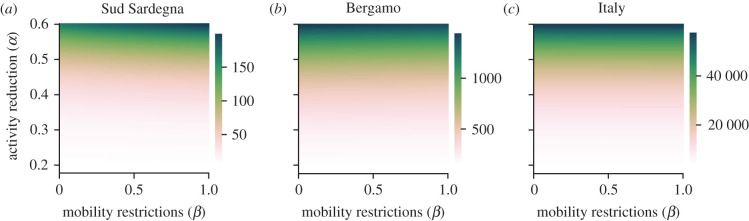
Effect of the relaxation of NPIs, for different levels of post-relaxation activity reduction and mobility restrictions, where higher levels of *α* or *β* denote less severe NPIs. We consider the number of deaths over a time window of 60 days from the relaxation date (18 May). In (*a*), we report the results for *Sud Sardegna*, in (*b*) for *Bergamo*, while in (*c*) we show the results aggregated at the country level.

Our results suggest that the effect of maintaining mobility restrictions after the relaxation of NPIs is negligible and dominated by the activity reduction (figure 8). We evaluate the total number of deaths in a time window of 60 days after the relaxation date (18 May). Both at the province level, for which we show the examples of *Sud Sargegna* (figure 8*a*) and *Bergamo* (figure 8*b*), and at the aggregated country level (figure 8*c*), the contribution of mobility restrictions is little or absent. These results are confirmed by other scenarios with different relaxation dates (electronic supplementary material, §§S5 and S6 and figures S3 and S4). Overall, this evidence indicates that activity reduction in the relaxation of NPIs should be thoughtfully calibrated, trading the risk of resurgent outbreaks against the social and economic costs associated with such policies. On the other hand, further enforcement of mobility restrictions within the country after the end of the epidemic wave does not seem to be beneficial in the relaxation phase.

## 4. Discussion

Motivated by the evidence of the key role of NPIs in the ongoing COVID-19 outbreak [2–6], we made an effort to propose a parsimonious mathematical framework to study NPIs and elucidate their impact on epidemic spreading. Specifically, we combined a meta-population model, capturing the spatial distribution of the population and its mobility patterns [23,24], with an ADN-based structure, which reflects real-world features of social activity, such as heterogeneity [29,30] and behavioural traits [27,28]. We explicitly incorporated two types of NPIs: actions aiming at reducing individuals’ activity (social distancing, forbidding gatherings and, in general, any measure that curtails the number of contacts favouring the spread of the infection) and policies to restrict individuals’ mobility (for instance, through travel bans). Through the lens of our modelling framework, we disentangled the effect of these two types of policies depending on the time of their implementation. We calibrated the model with data on the ongoing COVID-19 outbreak in Italy [32].

We leveraged the model to explore a wide range of what/if scenarios on the spatio-temporal dynamics of COVID-19 spreading for different combinations of NPIs. Our analysis allows us to draw interesting conclusions on when and how to apply NPIs to make the fight against the spread more effective. While the level of activity reduction is unequivocally a decisive factor, the impact of mobility restrictions has a more nuanced impact. First, we observed that mobility restrictions produce benefits only if applied at the early stage of the outbreak, and only if paired with appropriate activity reduction policies. Moreover, we discovered that the effect of mobility restrictions is strongly dependent on space. In fact, through a non-negative matrix factorization technique, we identified two sets of provinces that are differently affected by mobility restrictions. The first set, mostly consisting of provinces in the north (where the outbreak initially started), has little or no benefit from mobility restrictions. The provinces in the second set, instead, would have benefited from early implementation of mobility restrictions. Surprisingly, this set includes some of the provinces in the north (most affected area). Then, we discussed the possible implementation of targeted NPIs, with severe restrictions only for age cohorts that are more at risk of developing severe illness. Our modelling framework brought to light concerning limitations in the implementation of these targeted interventions: although economic reasons may prompt these interventions, their public health value could be limited. Finally, while mobility restrictions are useful in the early stage of the outbreak, their late implementation is ineffective. A different scenario is observed for the relaxation of NPIs, where the level of activity reduction should be carefully and gradually relaxed.

Our study outlines several avenues of future research, which can be pursued by leveraging the generality of the heterogeneous meta-population framework proposed in this study. During the first wave of COVID-19 in Italy, NPIs were homogeneously implemented nationwide through Decrees of the Prime Minister. Hence, we have used uniform parameters among the provinces. However, from November 2020, local NPIs have been enacted. The proposed model could benefit from the study of heterogeneous implementation (and relaxation) of NPIs between provinces and even the implementation of targeted mobility restrictions between specific provinces (through the modification of the routing matrix *W*), whose analysis is envisaged for future research. The outcome of such an analysis can inform policymakers on targeted interventions that may reduce social and economic costs while effectively halting the epidemic. Also, other targeted intervention policies, such as those aiming at reducing the activity of all high-risk individuals, regardless of their age, may be explored. Our model could also be used to assess strategies leading to safe reopening of schools. These studies may be conducted at the whole country level or at a local level. Country-wise interventions could be engineered by using further activity classes that capture, for instance, students and teachers. In this vein, a contact matrix among activity classes could help capture inhomogeneous interaction patterns between and within activity classes, similar to [40]. Local targeted interventions, instead, may be pursued via our meta-population structure, where communities are used to model specific locations, such as schools and neighbourhoods. The introduction of a community that represents the rest of the world would enable the study of the impact of closures of national borders.

While we considered a simple model for the progression of the epidemic, additional compartments and transitions may be added to capture hospitalization or testing [33] and used to analyse different what/if scenarios and design feedback control interventions, informed by the number of reported cases or hospitalizations [18]. A limitation of our modelling framework lies in its deterministic formulation, which prevents it from capturing phenomena such as local disease eradication. This may be crucial to study the pandemic at longer time scales, encompassing future vaccination campaigns. A stochastic formulation of the meta-population activity-driven model may be proposed and used as a viable tool to shed light onto these important phenomena and understand their impact on the spreading process. Finally, the simplicity of our mathematical framework may be conducive to a rigorous analytical treatment, involving, for example, the computation of the epidemic threshold, towards providing further insight into the effect of NPIs on the spread of the epidemic.

## Data accessibility

Epidemiological data are available at [32]; geographical, mobility and census data at [39]. The code used for the simulations is available at https://gitlab.com/PoliToComplexSystemLab/adn-metapopulation-2020.
